# Sintilimab-induced delayed-onset toxic epidermal necrolysis in a patient with gastric carcinoma: a case report and literature review

**DOI:** 10.3389/fonc.2026.1752915

**Published:** 2026-04-22

**Authors:** Qiaoqiao Cheng, Shilong Mao, Liming Wang, Xiaoyan Yang, Rongrong Zhang, Li Chen, Ling Jiang, Jinyin Li, Yiping Gao, Zi Ye

**Affiliations:** 1 Department of Pharmacy, Shanghai Xuhui Central Hospital, Shanghai, China; 2 Department of Oncology, Shanghai Xuhui Central Hospital, Shanghai, China

**Keywords:** complete response, gastric carcinoma, immune-related adverse event, neoadjuvant therapy, PD-1 inhibitor, sintilimab, toxic epidermal necrolysis

## Abstract

**Background:**

Sintilimab, a programmed death-1 (PD-1) inhibitor widely used in various malignancies, has demonstrated favorable antitumor efficacy and safety. However, severe immune-related cutaneous adverse events (irCAEs) such as toxic epidermal necrolysis (TEN) are rare but potentially fatal. Understanding their clinical characteristics, treatment strategies, and pathophysiological mechanisms is crucial for optimal management.

**Case presentation:**

We report a case of a patient with gastric carcinoma who developed delayed-onset TEN after receiving four cycles of neoadjuvant sintilimab combined with the SOX regimen (S-1 and oxaliplatin). The patient subsequently underwent radical gastrectomy, and postoperative pathology confirmed a complete pathological response. On the third postoperative day, the patient developed a scattered erythematous rash that began on the back and spread to the upper abdomen and upper and lower limbs, forming plaques. A dermatology consultation and clinical evaluation led to a diagnosis of immune-related TEN. The patient received systemic methylprednisolone and comprehensive supportive care, resulting in a gradual recovery of skin lesions, with no recurrence of TEN during follow-up.

**Discussion:**

This case suggests that sintilimab-induced TEN can occur as a delayed immune-mediated reaction, even after drug discontinuation. The potential mechanism of sintilimab-induced TEN may involve the excessive activation of cytotoxic T lymphocytes and dysregulated immune responses leading to keratinocyte apoptosis through Fas/FasL and perforin–granzyme pathways. Notably, the occurrence of irCAEs has been associated with favorable antitumor responses in some patients, reflecting strong immune activation. Prompt discontinuation of immunotherapy, early initiation of high-dose corticosteroids, and supportive measures remain the cornerstone of management. TNF-α or JAK inhibitors may provide therapeutic benefit in refractory cases.

**Conclusion:**

This case highlights a rare instance of delayed-onset TEN following sintilimab-based neoadjuvant therapy in gastric cancer, occurring shortly after surgical resection despite a complete pathological response. Early recognition and timely immunosuppressive treatment are essential for favorable outcomes. Further investigation into the mechanisms and predictive factors of PD-1 inhibitor–induced TEN is warranted.

## 1 Introduction

Gastric cancer (GC) remains a major global health burden, ranking as the fifth most common cancer worldwide. In 2022, approximately 968,000 new cases and 660,000 deaths were reported, with a disproportionately high incidence in East Asia, particularly China (1). Despite advancements in systemic therapies, the prognosis for locally advanced GC remains suboptimal, with 5-year survival rates below 30%. Neoadjuvant chemotherapy has become a cornerstone for resectable tumors, improving surgical outcomes and survival. Recently, immune checkpoint inhibitors (ICIs), particularly PD-1 inhibitors such as sintilimab, have demonstrated remarkable efficacy when combined with chemotherapy, leading to higher rates of pathological complete response (pCR) and long-term survival benefits in clinical trials (2). However, the potent immunostimulatory effects of ICIs are accompanied by an increased risk of immune-related adverse events (irAEs). Among these, cutaneous irAEs are the most common, occurring in approximately 30–50% of treated patients.

Although the majority of cutaneous irAEs are manageable, Stevens-Johnson syndrome (SJS) and toxic epidermal necrolysis (TEN) are rare but potentially fatal reactions (3). TEN is characterized by extensive epidermal necrosis and detachment involving more than 30% of the body surface area (BSA).

Herein, we present a case of delayed-onset TEN in a patient with gastric cancer who achieved complete pathological remission following neoadjuvant sintilimab plus SOX therapy. This case highlights the importance of extended monitoring for delayed irAEs even after treatment completion and provides mechanistic insights into PD-1 inhibitor–related severe cutaneous toxicity through clinical observation and literature review.

## 2 Case presentation

The patient, a 54-year-old male weighing 65 kg with a body mass index (BMI) of 22.49, was diagnosed with gastric cancer in November 2023. The patient and his family members had no history of immune-related diseases. Histopathological examination of gastric biopsy specimens demonstrated adenocarcinoma of the gastric body, Lauren mixed type, grade II–III. Immunohistochemical analysis revealed a Ki-67 index of approximately 30%. Immunohistochemical results were as follows: HER-2 (40%+, 1+), MSH2 (100%+++), MSH6 (100%+++), PMS2 (100%+), MLH1 (tumor -, stroma -), Claudin18.2 (-), c-MET (100%++), PD-L1 (tumor -, stroma 1%+), and EBER (+). PD-L1 testing suggested potential benefit from PD-1/PD-L1 ICIs. After further examination at our hospital and exclusion of contraindications, the patient was initiated on neoadjuvant chemotherapy combined with immunotherapy. The regimen consisted of the SOX protocol (oxaliplatin 230 mg d1 + S-1 60 mg, po, bid, d1–14) combined with the PD-1 inhibitor sintilimab (200 mg, d2, every 3 weeks) for four cycles, administered between December 2023 and February 2024. On March 14, 2024, the patient underwent radical gastrectomy for gastric cancer, and the resected specimen (post-neoadjuvant therapy for gastric adenocarcinoma) was sent for examination. Pathological evaluation after neoadjuvant therapy showed a complete response. The patient’s perioperative medication regimen included anti-infective prophylaxis (cefazoxime), acid-suppressive therapy (ranitidine), and analgesia and anesthesia (propacetamol, nalmefene, remifentanil, ropivacaine, sevoflurane, rocuronium, and dexmedetomidine).

**Figure 1:**
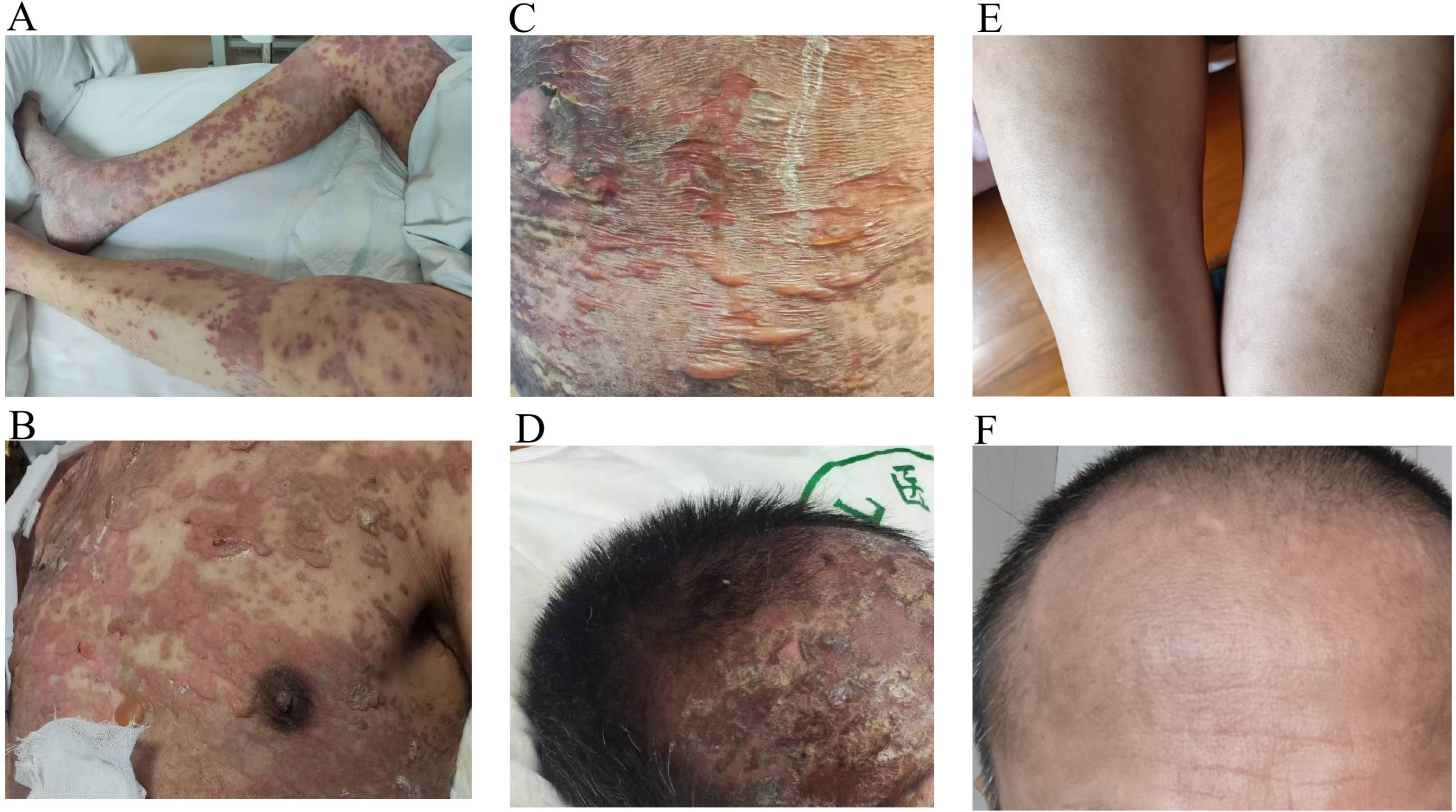
Progression of the dermatological condition over time. **(A)** Cutaneous symptoms at 3 days postoperatively. **(B)** Cutaneous symptoms at 10 days postoperatively (pretreatment). **(C)** Cutaneous symptoms at 1 month postoperatively. **(D)** Cutaneous symptoms at 2 months postoperatively (posttreatment). **(E, F)** Cutaneous symptoms during recovery.

Three days postoperatively, the patient developed scattered erythematous rashes starting on the back and spreading to the upper abdomen and upper and lower limbs, forming plaques (**Figure 1A**). The lesions were painful, pruritic, and associated with blistering and oral mucosal ulcerations. Initial conservative management with intravenous vitamin C, topical epidermal growth factor, Compound Huangbai lotion for affected skin, calamine lotion for non-eroded areas, oral Kangfuxin liquid for oral ulcers, and antihistamines (levocetirizine and ebastine) plus compound glycyrrhizin failed to improve the condition. The rash worsened, with fluid exudation and rapidly expanding bullae, affecting approximately 80% of the body surface area (**Figure 1B**). Following dermatology consultation, physical examination confirmed a positive Nikolsky sign (**Figure 1C**), characterized by epidermal detachment upon gentle lateral pressure applied to clinically normal skin adjacent to the lesions. Histopathological examination was not performed, primarily due to the patient’s critical clinical status (with treatment and resuscitation prioritized) and refusal by the patient and his family, who expressed concerns regarding exacerbated pain and increased financial burden associated with the procedure. A comprehensive differential diagnosis was conducted to rule out SJS, staphylococcal scalded skin syndrome (SSSS), autoimmune bullous diseases, SJS/TEN induced by conventional drugs, and erythroderma. Combined with the patient’s medication history, typical clinical manifestations, and exclusion of other etiologies, the patient was clinically diagnosed with immune-related TEN. The rash was onset occurred on March 17 and progressed to involve ≥80% of BSA by March 24, with a progression time of seven days. The severity-of-illness score for toxic epidermal necrolysis (SCORTEN) scale, a validated tool for assessing TEN severity and prognosis, was applied, yielding a score of 3 points. The scoring criteria included age >40 years, a history of cancer, and BSA involvement >10%, corresponding to an estimated in-hospital mortality risk of 35%. High-dose intravenous methylprednisolone (60 mg) was initiated, accompanied by supportive care including anti-infection, hepatoprotective therapy, and electrolyte management. Over the following month, the exudation decreased, some lesions began to crust, and pain was alleviated. Corticosteroid therapy was gradually tapered to 30 mg daily and then further to 10 mg over subsequent weeks. Two months later, existing vesicles and erythema had subsided, with skin crusting, drying, and peeling observed, and no new rashes appeared (**Figure 1D**), showing marked improvement compared with the pretreatment condition (**Figure 1B**).

**Figure 2:**
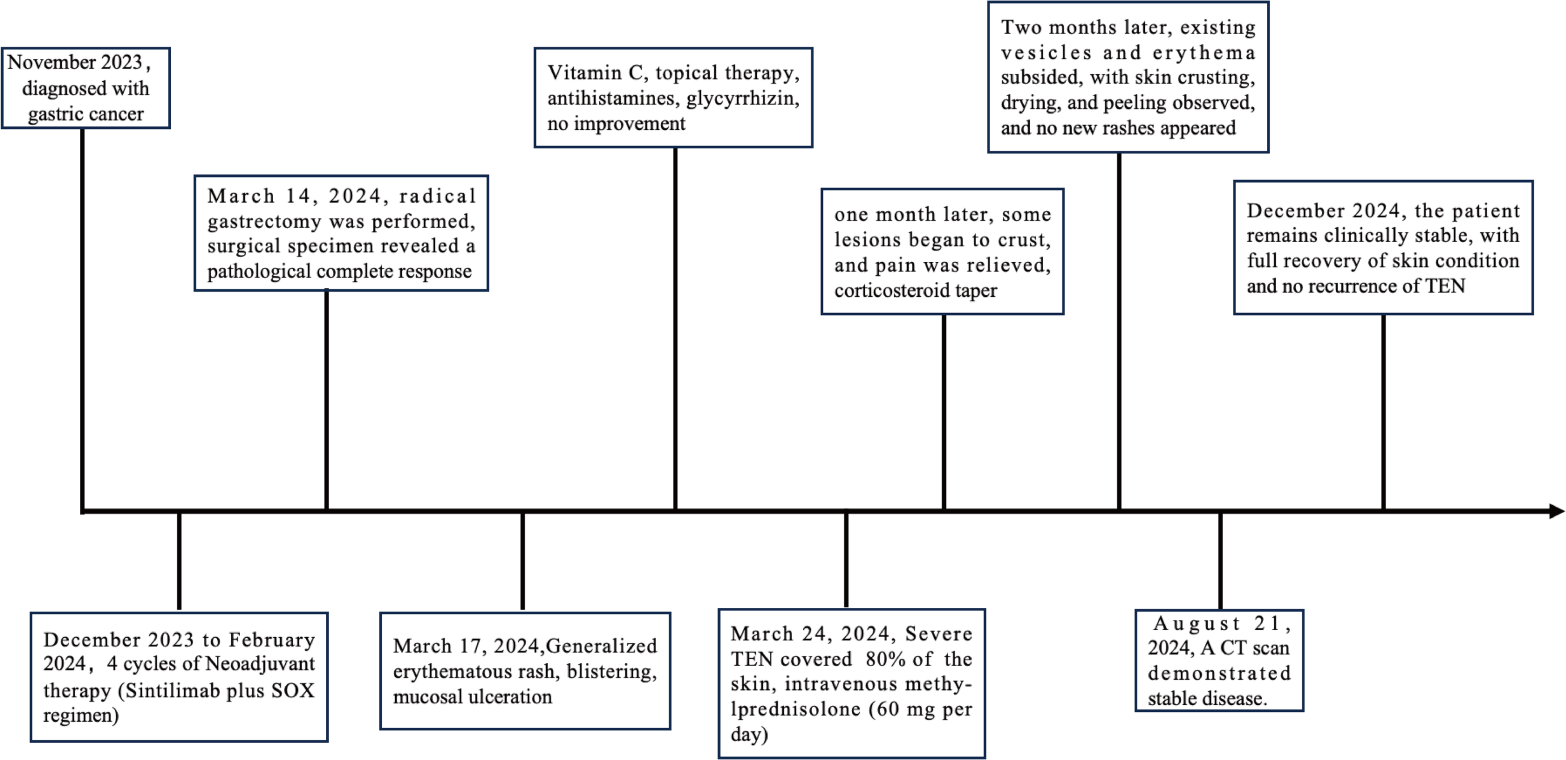
Treatment timeline.

Postoperatively, the patient received two cycles of adjuvant chemotherapy (oxaliplatin 200 mg d1 + S-1 60 mg, po, bid, d1–14, Q3W) from May to June 2024. Due to repeated bone marrow suppression and gastrointestinal toxicity, treatment was switched to maintenance S-1 monotherapy (60 mg, po, bid, d1–14, q3w). A CT scan on August 21, 2024, demonstrated stable disease. As of December 2024, the patient remains clinically stable, with full recovery of skin condition and no recurrence of TEN (**Figures 1E, F**). The treatment timeline is presented in **Figure 2**.

## 3 Discussion

In this case report, the GC patient received four cycles of neoadjuvant therapy with sintilimab combined with the SOX regimen, followed by radical gastrectomy. Postoperative pathological evaluation showed a complete response. However, three days postoperatively, the patient developed a scattered erythematous rash, which later progressed to TEN. The TEN affected approximately 80% of the body surface area. Previous reports have documented sintilimab-induced TEN (3), but it is relatively rare for TEN to occur after achieving pCR following neoadjuvant therapy. Immune checkpoint inhibitors can cause immune-related adverse events that may manifest long after treatment discontinuation. There are no reports of oxaliplatin directly causing TEN. The patient underwent adjuvant therapy with S-1 maintenance treatment postoperatively, without significant adverse reactions. To rule out the possibility of perioperative drug-induced reactions and evaluate the causality between sintilimab and TEN, we performed a causality assessment for all suspected drugs using the Algorithm of Drug Causality for Epidermal Necrolysis (ALDEN) scale. Sintilimab scored two points and was classified as a possible cause of delayed-onset TEN, while nalmefene and rocuronium were assessed as unlikely triggers. All other perioperative medications were categorized as very unlikely to be associated with this reaction. These findings suggest that sintilimab remains the most plausible candidate agent responsible for TEN in this patient, despite the confounding surgical context.

The patient developed TEN more than 3 months after the initial administration of sintilimab. A systematic review, which included 50 cancer patients who developed SJS/TEN following immune checkpoint inhibitor (ICI) therapy, revealed that SJS/TEN typically occurs early, with a median onset of 23 days post-ICI initiation (3). However, evidence suggests that TEN onset can be delayed. A 2022 case report described a patient with NK/T-cell lymphoma who developed extensive rashes that progressed rapidly to TEN after four cycles of gemcitabine plus sintilimab immunotherapy (4). In our report, the patient, despite achieving pCR after four cycles of neoadjuvant sintilimab combined with the SOX regimen, still developed TEN. This underscores the necessity for vigilance regarding TEN risk even during advanced treatment stages.

According to the National Comprehensive Cancer Network (NCCN) Guidelines Version 1.2025 for the Management of Immunotherapy-Related Toxicities, when SJS or TEN occur, urgent dermatology consultation, intravenous methylprednisolone (1–2 mg/kg/day), and consideration of intravenous immunoglobulin (IVIG) are recommended. Notably, the initial methylprednisolone dose (60 mg, 0.92 mg/kg/day) administered to this 65-kg patient was lower than the recommended 1–2 mg/kg/day for TEN. This conservative strategy was chosen due to the patient’s recent radical gastrectomy and elevated infection risk. However, this case underscores the importance of early and adequate glucocorticoid therapy in ICI-related TEN, and insufficient initial dosing may represent a clinical lesson for future similar cases. Although this patient developed sintilimab-associated TEN, pCR was achieved after four cycles of neoadjuvant therapy. The raises the question: are cutaneous irAEs associated with improved prognosis in patients receiving ICI treatment?

Multiple high-quality systematic reviews and meta-analyses support a positive correlation between cutaneous adverse events and ICI efficacy. A 2023 meta-analysis encompassing 23 studies (22,749 patients) demonstrated that the development of cutaneous irAEs (cirAEs) was significantly associated with improved overall survival (OS) and progression-free survival (PFS) (5). Similarly, another study found that the incidence of cirAEs positively correlated with both response rate and PFS. Specifically, the incidence of pruritus, vitiligo, and rash correlated with increased response rates, while vitiligo occurrence showed a statistically significant association with improved OS (6). Based on data from the TriNetX Diamond Network including 7,008 patients, the occurrence of cirAEs, including pruritus, rash, xerosis, psoriasis, and lichenoid dermatitis, was associated with a significantly reduced risk of death (7). A single-center retrospective study involving 174 melanoma patients treated with PD-1 inhibitors also reported significantly improved PFS and OS in patients who developed cutaneous adverse events compared with those who did not develop cutaneous toxicity (8).

Despite substantial evidence supporting an association between cirAEs and improved efficacy, inconsistent findings and ongoing controversy remain, suggesting that this relationship may be influenced by multiple factors (9). Furthermore, most supportive evidence originates from retrospective studies and meta-analyses, which inherently carry risks of selection bias and confounding. For instance, patients with longer survival have more opportunities to develop and report adverse events. Additionally, factors such as tumor type, ICI agent class (anti-CTLA-4 vs. anti-PD-1/PD-L1), combination regimens (e.g., chemotherapy or targeted therapy), and patient characteristics (e.g., age, weight, and comorbidities) may influence both the incidence and type of cirAEs and may act as confounders affecting efficacy outcomes (10, 11). This complexity makes it difficult to determine the independent prognostic value of cirAEs. Therefore, more rigorously designed prospective studies are needed to establish a causal relationship between cirAEs and treatment efficacy. Current research has primarily focused on grade 1–3 cutaneous adverse reactions. The correlation between rare but severe grade 4 cutaneous reactions (such as SJS/TEN) and treatment efficacy remains largely unexplored. It is still unclear whether these severe reactions are associated with improved efficacy or, conversely, negatively impact prognosis due to the necessity of treatment interruption (11, 12).

TEN is a rare but life-threatening severe cutaneous adverse reaction with a complex pathogenesis that is not yet fully understood. Substantial evidence suggests that the core mechanism of TEN involves drug-specific cytotoxic T lymphocytes (CTLs) mediating attacks against keratinocytes (13, 14). Sintilimab, as a PD-1 inhibitor, exerts its therapeutic effect by blocking the PD-1/PD-L1 pathway, thereby releasing the suppression on T cells and enhancing antitumor immune responses. However, this non-specific immune activation may also disrupt immune tolerance, leading T cells to attack self-tissues, including skin keratinocytes, thereby triggering TEN (3, 15). Regarding how CTLs kill keratinocytes, multiple theories have been proposed, some of which remain controversial; theories suggest synergistic actions of various molecules and signaling pathways. Key effector molecules released by CTLs and natural killer (NK) cells, such as granzymes (particularly granzyme B) and granulysin, mediate keratinocyte death (16). The Fas/Fas ligand (FasL) signaling pathway also participates in amplifying apoptosis, while cytokines, such as tumor necrosis factor-α (TNF-α) and interferon-γ (IFN-γ), enhance the sensitivity of keratinocytes to apoptotic signals through upstream regulation (17). The activation of interferon (IFN) signaling pathways and JAK/STAT pathways plays a critical driving role in the pathogenesis of TEN. Therefore, clinical research progress targeting these pathways should be closely monitored, as this may offer new directions for future treatment. In certain refractory cases, TNF-α inhibitors (such as adalimumab) may be effective. For example, a metastatic gastric cancer patient who developed TEN after sintilimab treatment showed no response to corticosteroids or IVIG, but the rash rapidly improved within 24 hours after receiving the TNF-α inhibitor adalimumab (18). A 2024 study explored the therapeutic potential of JAK inhibitors. The research reported good safety and rapid skin recovery in seven TEN patients treated with JAK inhibitors (such as tofacitinib and baricitinib), offering a novel direction for TEN therapy (19).

In the analysis of the FDA Adverse Event Reporting System (FAERS), PD-1 inhibitors demonstrated a strong signal for SJS/TEN overall (20). Heterogeneity in SJS/TEN signals was observed across different immune checkpoint inhibitors (ICIs), with pembrolizumab showing the strongest signals for both SJS (ROR = 4.36) and TEN (ROR = 5.49). The median onset of PD-1-related SJS/TEN was approximately 25.5 days (21.5 days for SJS and 32 days for TEN) (21). As a PD-1 inhibitor, sintilimab-induced TEN exhibited a latency consistent with the general pattern of PD-1/PDL-1 class agents but a significantly delayed onset compared with non-ICI drugs. Compared with TEN caused by non-ICI agents, ICI-related TEN typically involves a smaller body surface area and milder oral and ocular mucosal involvement (22). The immunopathological mechanism underlying sintilimab-induced TEN is largely consistent with that of other PD-1inhibitors (e.g., nivolumab and tislelizumab), involving disruption of T-cell homeostasis and keratinocyte damage following PD-1/PD-L1 blockade (23, 24). Moreover, such PD-1 inhibitor-induced TEN generally responds well to the combined regimen of systemic glucocorticoids (e.g., methylprednisolone) and IVIG therapy (15, 24). In monotherapy settings, the sintilimab group has been associated with a higher incidence of TEN, larger affected body surface area, and longer time to re-epithelialization compared with other PD-1 inhibitors, despite similar overall mortality rates (3). Another real-world study reported that the overall incidence of sintilimab-related irAEs was 55.56%, significantly higher than that of pembrolizumab (38.98%) and toripalimab (33.33%) (25).

We compiled a table (**Table 1**) reviewing the literature on sintilimab-related rashes (15, 18, 26–30). Although some of these studies partially overlap with our findings, this case is clinically rare, as the patient developed TEN after achieving a complete response following neoadjuvant sintilimab therapy. We provided a detailed description of the clinical manifestations, treatment, and outcomes, and discussed the characteristics of SJS/TEN induced by sintilimab, the association between ICI-related cutaneous adverse events and clinical outcomes, along with the underlying mechanisms of TEN. This report serves as a warning and reference for the clinical use of sintilimab and the management of cutaneous adverse events.

**Table 1:** Comparison of sintilimab-related rash cases: a review of existing literature.

Number	Author, Year	Cancer type	Regimen	Onset timing	Therapy	Re-epithelization time
1	Yang et al., 2022 (15)	Thymic carcinoma	Sintilimab	3 months	Methylprednisolone immunoglobulin	1 month
2	Zhang et al., 2023 (18)	Gastric malignancy	Sintilimab, oxaliplatin, and tiggio	10 days	Methylprednisolone immunoglobulin adalimumab	45 days
3	Zhao et al., 2022 (26)	Gallbladder carcinoma	Sintilimab and anlotinib	2 weeks	Methylprednisolone immunoglobulin	43 days
4	Li et al., 2022 (27)	Non-small cell lung cancer	Sintilimab and conventional chemotherapy	5 months	Methylprednisolone prednisone	2 months
5	Li et al., 2021 (28)	Cervical tumor	Paclitaxel, nedaplatin, and sintilimab	70 days	Diphenhydramine methylprednisolone immunoglobulins	Died
6	Gong et al., 2023 (29)	Intrahepatic cholangiocarcinoma.	Sintilimab and lenvatinib	18 days	Steroids immunoglobulin	Died
7	Li et al., 2023 (30)	Lung adenocarcinoma	Sintilimab and paclitaxel liposome	9 weeks	Methylprednisolone immunoglobulin	46 days

## Data Availability

The datasets presented in this study can be found in online repositories. The names of the repository/repositories and accession number(s) can be found in the article/supplementary material.

## References

[1] Bray F Laversanne M Sung H Ferlay J Siegel RL Soerjomataram I . Global cancer statistics 2022: GLOBOCAN estimates of incidence and mortality worldwide for 36 cancers in 185 countries. Ca-Cancer J Clin. (2024) 74:229–63. doi: 10.3322/caac.21834. PMID: 38572751

[2] Huang X Fang J Huang L Chen H Chen H Chai T . SOX combined with sintilimab versus SOX alone in the perioperative management of locally advanced gastric cancer: a propensity score-matched analysis. Gastric Cancer. (2023) 26:1040–50. doi: 10.1007/s10120-023-01431-z. PMID: 37768447 PMC10640399

[3] Zhou J Wang C Li J Zhang H He C . Stevens-Johnson syndrome and toxic epidermal necrolysis associated with immune checkpoint inhibitors: a systematic review. Front Immunol. (2024) 15:1414136. doi: 10.3389/fimmu.2024.1414136. PMID: 39072330 PMC11272453

[4] Yang W Xu X Xia D Wang H Jiang J Yang G . Toxic epidermal necrolysis associated with chemoimmunotherapy for lymphoma: case report and literature review. Immunotherapy-Uk. (2022) 14:275–82. doi: 10.2217/imt-2021-0074. PMID: 35128931

[5] Du Y Wu W Chen M Dong Z Wang F . Cutaneous adverse events and cancer survival prognosis with immune checkpoint inhibitor treatment: a systematic review and meta-analysis. JAMA Dermatol. (2023) 159:1093–101. doi: 10.1001/jamadermatol.2023.3003. PMID: 37672255 PMC10483383

[6] Curkovic NB Bai K Ye F Johnson DB . Incidence of cutaneous immune-related adverse events and outcomes in immune checkpoint inhibitor-containing regimens: a systematic review and meta-analysis. Cancers. (2024) 16:340. doi: 10.3390/cancers16020340. PMID: 38254829 PMC10814132

[7] Tang K Seo J Tiu BC Le TK Pahalyants V Raval NS . Association of cutaneous immune-related adverse events with increased survival in patients treated with anti-programmed cell death 1 and anti-programmed cell death ligand 1 therapy. JAMA Dermatol. (2022) 158:189–93. doi: 10.1001/jamadermatol.2021.5476. PMID: 35019948 PMC8756357

[8] Pozsgai M Sebastian UF Olah P Nemeth V Gyulai R Lengyel Z . Cutaneous side effects of PD-1 inhibitors: a single-center retrospective study. Int J Dermatol. (2025) 64:1066–78. doi: 10.1111/ijd.17683. PMID: 39925013 PMC12082620

[9] Han Y Wang J Xu B . Cutaneous adverse events associated with immune checkpoint blockade: a systematic review and meta-analysis. Crit Rev Oncol Hemat. (2021) 163:103376. doi: 10.1016/j.critrevonc.2021.103376. PMID: 34087346

[10] Nikolaou VA Apalla Z Carrera C Fattore D Sollena P Riganti J . Clinical associations and classification of immune checkpoint inhibitor-induced cutaneous toxicities: a multicentre study from the European Academy of Dermatology and Venereology Task Force of Dermatology for Cancer Patients. Brit J Dermatol. (2022) 187:962–9. doi: 10.1111/bjd.21781. PMID: 35861701

[11] Esen BH Ozbek L Oguz S Selcukbiricik F . Characterizing immune checkpoint inhibitor-related cutaneous adverse reactions: a comprehensive analysis of FDA adverse event reporting system (FAERS) database. Heliyon. (2024) 10:e33765. doi: 10.1016/j.heliyon.2024.e33765. PMID: 39071598 PMC11283008

[12] Godfrey H Jedlowski P Thiede R . Severe cutaneous adverse reactions associated with the immune checkpoint inhibitors: a case/non-case analysis using the Food and Drug Administration Adverse Event Reporting System. Australas J Dermatol. (2024) 65:243–53. doi: 10.1111/ajd.14262. PMID: 38572842

[13] Schwartz RA McDonough PH Lee BW . Toxic epidermal necrolysis: part I. Introduction, history, classification, clinical features, systemic manifestations, etiology, and immunopathogenesis. J Am Acad Dermatol. (2013) 69:171–173, 185-186. doi: 10.1016/j.jaad.2013.05.003. PMID: 23866878

[14] Borchers AT Lee JL Naguwa SM Cheema GS Gershwin ME . Stevens-Johnson syndrome and toxic epidermal necrolysis. Autoimmun Rev. (2008) 7:598–605. doi: 10.1016/j.autrev.2008.06.004. PMID: 18603022

[15] Yang H Ma Q Sun Y Zhang K Xing Y Li H . Case report: toxic epidermal necrolysis associated with sintilimab in a patient with relapsed thymic carcinoma. Front Oncol. (2022) 12:1065137. doi: 10.3389/fonc.2022.1065137. PMID: 36620577 PMC9813861

[16] Chung W Hung S Yang J Su S Huang S Wei C . Granulysin is a key mediator for disseminated keratinocyte death in Stevens-Johnson syndrome and toxic epidermal necrolysis. Nat Med. (2008) 14:1343–50. doi: 10.1038/nm.1884. PMID: 19029983

[17] Viard-Leveugle I Gaide O Jankovic D Feldmeyer L Kerl K Pickard C . TNF-alpha and IFN-gamma are potential inducers of Fas-mediated keratinocyte apoptosis through activation of inducible nitric oxide synthase in toxic epidermal necrolysis. J Invest Dermatol. (2013) 133:489–98. doi: 10.1038/jid.2012.330. PMID: 22992806

[18] Zhang L Wu Z . Adalimumab for sintilimab-induced toxic epidermal necrolysis in a patient with metastatic gastric Malignancy: a case report and literature review. Clin Cosmet Inv Derm. (2023) 16:457–61. doi: 10.2147/CCID.S401286. PMID: 36846442 PMC9951597

[19] Nordmann TM Anderton H Hasegawa A Schweizer L Zhang P Stadler P . Spatial proteomics identifies JAKi as treatment for a lethal skin disease. Nature. (2024) 635:1001–9. doi: 10.1038/s41586-024-08061-0. PMID: 39415009 PMC11602713

[20] Lu W Zhang H Guo Q Gou Z Yao J . Selected cutaneous adverse events in patients treated with ICI monotherapy and combination therapy: a retrospective pharmacovigilance study and meta-analysis. Front Pharmacol. (2023) 14:1076473. doi: 10.3389/fphar.2023.1076473. PMID: 37332342 PMC10272362

[21] Zhu J Chen G He Z Zheng Y Gao S Li J . Stevens-Johnson syndrome/toxic epidermal necrolysis in patients treated with immune checkpoint inhibitors: a safety analysis of clinical trials and FDA pharmacovigilance database. Eclinicalmedicine. (2021) 37:100951. doi: 10.1016/j.eclinm.2021.100951. PMID: 34386743 PMC8343267

[22] Qin K Gong T Ruan S Lin M Su X Lv X . Clinical features of Stevens-Johnson syndrome and toxic epidermal necrolysis induced by immune checkpoint inhibitor versus non-immune checkpoint inhibitor drugs in China: a cross-sectional study and literature review. J Inflammation Res. (2024) 17:7591–605. doi: 10.2147/JIR.S491791. PMID: 39464339 PMC11512543

[23] Li R Lei H Wang C Liu X . Clinical features of nivolumab-induced Stevens-Johnson syndrome/toxic epidermal necrolysis: retrospective analysis based on case reports. Front Immunol. (2025) 16:1563100. doi: 10.3389/fimmu.2025.1563100. PMID: 40170847 PMC11958938

[24] Jin S Liu Z Zheng F . Tislelizumab-associated toxic epidermal necrolysis in an esophageal cancer patient: a case report. Front Immunol. (2025) 16:1707956. doi: 10.3389/fimmu.2025.1707956. PMID: 41268547 PMC12626797

[25] Huang G Liu S Dong J Xi X Kong R Li W . PD-1 inhibitor-based adverse events in solid tumors: a retrospective real-world study. Front Pharmacol. (2022) 13:974376. doi: 10.3389/fphar.2022.974376. PMID: 36438818 PMC9681783

[26] Zhao Y Cao Y Wang X Qian T . Treatment of PD-1 inhibitor-associated toxic epidermal necrolysis: a case report and brief review. Oncotargets Ther. (2022) 15:345–51. doi: 10.2147/OTT.S353743. PMID: 35422628 PMC9005125

[27] Li G Gong S Wang N Yao X . Toxic epidermal necrolysis induced by sintilimab in a patient with advanced non-small cell lung cancer and comorbid pulmonary tuberculosis: a case report. Front Immunol. (2022) 13:989966. doi: 10.3389/fimmu.2022.989966. PMID: 36090976 PMC9459224

[28] Li X Qu L Ren Y Hu C . Case report: a case report and literature review on severe bullous skin reaction induced by anti-PD-1 immunotherapy in a cervical cancer patient. Front Pharmacol. (2021) 12:707967. doi: 10.3389/fphar.2021.707967. PMID: 34504425 PMC8423354

[29] Gong Y Mao J Liu M Gao J . A case of toxic epidermal necrolysis associated with lenvatinib and sintilimab therapy for intrahepatic cholangiocarcinoma. J Int Med Res. (2023) 51:655732812. doi: 10.1177/03000605231173556. PMID: 37211771 PMC10204045

[30] Li X Li G Chen D Su L Wang R Zhou Y . Case report: sintilimab-induced Stevens-Johnson syndrome in a patient with advanced lung adenocarcinoma. Front Oncol. (2023) 13:912168. doi: 10.3389/fonc.2023.912168. PMID: 37781182 PMC10540079

